# Formulation-dependent kinetics of *Lacticaseibacillus paracasei* Zhang in mice

**DOI:** 10.1128/spectrum.03637-25

**Published:** 2026-04-21

**Authors:** Ni Han, Fan Bai, Qian Wen, Yujing Bi, Ruifu Yang, Yanping Han

**Affiliations:** 1 State Key Laboratory of Pathogen and Biosecurity, Academy of Military Medical Sciences 71040, Beijing, China; 2 School of Public Health, Key Laboratory of Environmental Pollution Monitoring and Disease Control, Ministry of Education, Guizhou Medical University 74628, Guiyang, China; Yangzhou University, Yangzhou, Jiangsu, China

**Keywords:** probiotic, *L. paracasei *Zhang, kinetics, gut microbiota, uric acid

## Abstract

The relationship between gut microbiota and human health has become one of the focal point in medical research. Probiotics, which modulate the gut microbiome, hold considerable promise for both prophylaxis and therapeutic intervention. This requires deeper insights into the kinetic changes and molecular mechanisms upon probiotic entry into the body. In this study, we utilized advanced molecular imaging to delineate the *in vivo* kinetic dynamics of two *Lacticaseibacillus paracasei* Zhang (*L. paracasei* Zhang, LPZ) formulations: a liquid culture and a lyophilized powder. Our results provide new insights into the gastrointestinal transit and growth kinetics of the different probiotics formulations. Strikingly, the liquid LPZ achieved its peak growth phase within a relatively short period of 6 to 8 h post-ingestion, culminating in a 270- to 680-fold increase in residues at the 24th hour post-ingestion when compared to the lyophilized powder LPZ. Furthermore, during peak *in vivo* replication, LPZ enhanced gut microbial diversity and enriched beneficial commensal communities. Functionally, LPZ ingestion attenuated virulence factors while upregulating carbohydrate-active enzymes. Notably, LPZ significantly reduced xanthine levels, a metabolite associated with hyperuricemia, thereby providing a mechanistic basis for the observed relief from gout symptoms. This supports the mechanism of prior clinical findings and paves the way for future clinical trials and therapeutic use of LPZ and related probiotics.

**IMPORTANCE:**

The innovation of this study lies in visualizing the kinetic changes of two *Lacticaseibacillus paracasei* Zhang (*L. paracasei* Zhang, LPZ) formulations (a liquid culture and lyophilized powder) within the gastrointestinal tract. It was found that liquid LPZ proliferates *in vivo* with a higher retention rate. Furthermore, we also found that when liquid LPZ reaches its peak proliferation phase *in vivo*, it not only effectively promotes the proliferation of other beneficial bacteria and the production of their metabolites but also generates more carbohydrate-active enzymes while reducing virulence factors, thereby amplifying the functions of LPZ. Meanwhile, we observed that liquid LPZ significantly reduces the production of xanthine *in vivo*, indicating its potential to lower uric acid. In light of the aforementioned findings, we herein propose the concept of “probiotikinetics.” These results provide new insights into the intake of LPZ, along with important evidence for its application in healthy populations.

## INTRODUCTION

The human gut microbiota constitutes a highly complex and dynamic ecosystem intricately involved in various physiological processes (1, 2). Probiotics, officially defined as “live microorganisms that, when administered in adequate amounts, confer a health benefit on the host” (3, 4), have garnered significant scientific and commercial interest due to their potential to modulate the gut microbiota and improve health. Despite the widespread application of probiotics in functional foods and clinical adjuvant therapy, significant gaps remain in our understanding of their *in vivo* mechanism of action (5–7). A major challenge in probiotic research is the limited understanding of their specific dynamics within the gut post-ingestion. Although probiotics must survive the harsh gastrointestinal environment to exert beneficial effects (8), the precise routes and mechanisms by which they navigate and colonize remain unclear. This uncertainty hampers the rational design of probiotic formulations and optimal ingestion strategies, thereby limiting their full therapeutic potential.

In response to these challenges, extensive research has focused on developing external encapsulation delivery systems for probiotics (9–11). However, the methods used to construct such delivery systems may compromise probiotics viability. For instance, encapsulation processes may hinder the timely release of beneficial metabolites produced by probiotics, thereby reducing their efficiency compared to free-form probiotics (12). Notably, the differential effects of high-viability liquid probiotics and dormant lyophilized powder probiotics (used as “biotherapeutics”) administered in an unencapsulated state remain unclear. This is particularly true regarding their release kinetics and bacterial load dynamics within the gastrointestinal tract.

This study aims to monitor the maintenance of probiotic vitality by comprehensively comparing the *in vivo* effects of liquid and lyophilized powder probiotics. Using LPZ (13) as a model strain, we demonstrated formulation-dependent patterns in its *in vivo* behavior. Our results reveal that the ingestion of LPZ enhances the diversity of the gut microbiota and promotes the enrichment of the resident probiotic communities. Furthermore, we introduce the new interdisciplinary concept of “Probiotic Kinetics.” Traditionally, the evaluation of probiotics has relied on the pharmacokinetics framework (14), focusing primarily on “Absorption, Distribution, Metabolism, and Excretion” (ADME) processes. However, this theory, which is based on chemical drugs, proves inadequate for describing living microorganisms. Probiotics are not inert molecules but “living biotherapeutic agents” capable of metabolism, replication, and dynamic crosstalk with their environment (4). Consequently, their therapeutic efficacy relies not merely on survival, but more fundamentally on the dynamic, bidirectional interactions established with the host and the indigenous gut microbiota (15). Thus, the establishment of a specialized theoretical framework “Probiotic Kinetics” is imperative. Probiotic Kinetics extends beyond the traditional ADME model by encompassing three core dimensions: organismal kinetics (governing distribution, retention, and clearance within the gastrointestinal tract), effect kinetics (pertaining to direct or indirect biological impacts on the host), and, most significantly, interaction kinetics (characterizing real-time signaling and metabolic exchange between probiotics and host cells, the immune system, and the native microbiota). Emerging research on probiotic-host metabolic interactions offers compelling validation for this paradigm. Notably, metabolites derived from specific probiotic strains (e.g., short-chain fatty acids [16, 17], neurotransmitter precursors [18]) function as signaling molecules that directly modulate host gene expression and metabolic pathways and even influence behavior via the gut-brain axis (19). These observations underscore that probiotic efficacy is fundamentally a profound “metabolic dialog” with the host, in which the timing and efficiency of initiating this dialog dictate the ultimate therapeutic outcomes.

## RESULTS

### Formulation-dependent kinetics of LPZ in mice

#### Construction of anaerobic fluorescent probiotics LPZ-pp1

**Fig 1:**
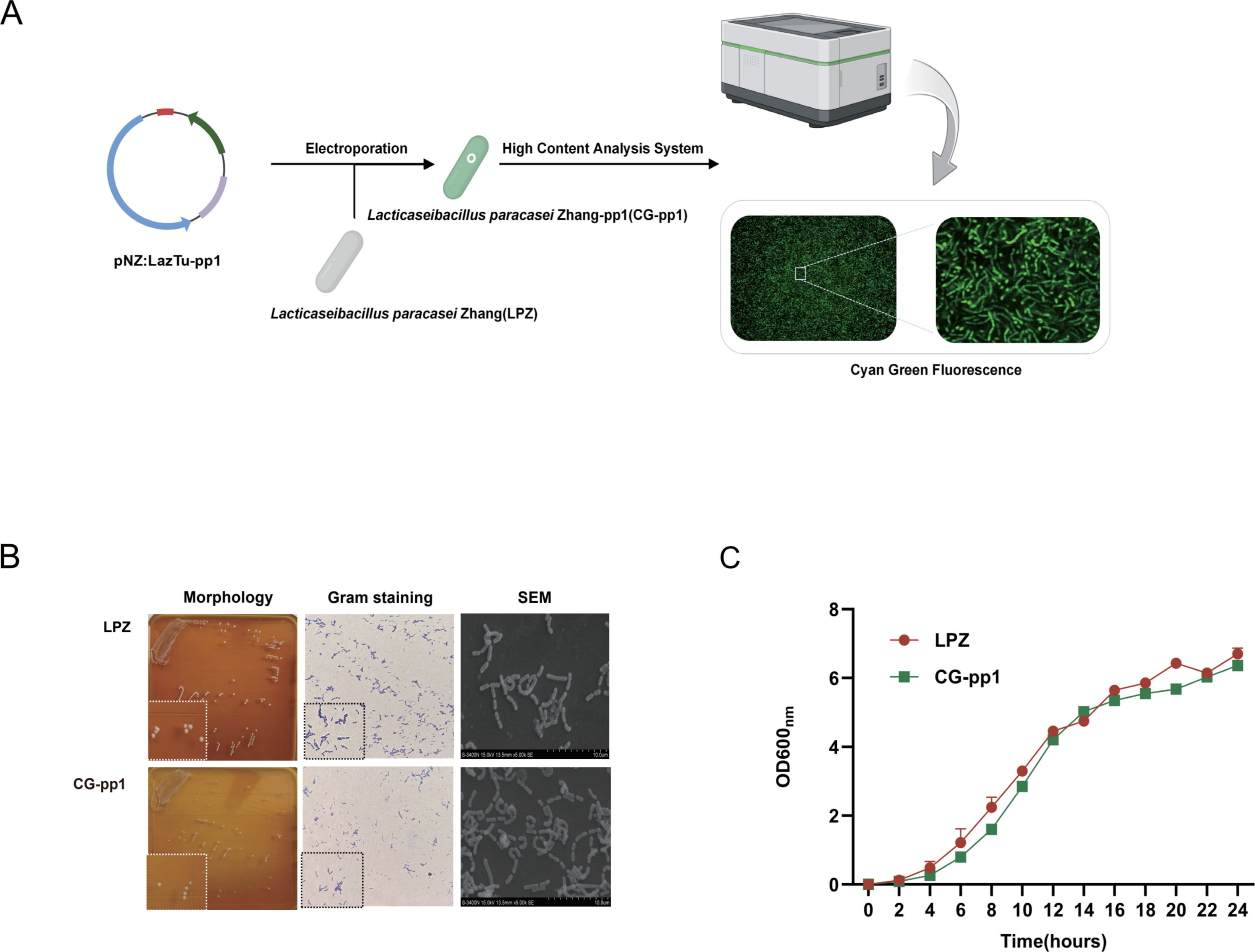
Construction of anaerobic fluorescent probiotics LPZ. (**A**) Study design and flow diagram. Construction and Verification of CG-pp1. The objective of this construction was to engineer CG-pp1 to emit turquoise light. (**B**) Morphological comparison of CG-pp1 and LPZ: Phenotype, Gram staining, and scanning electron microscopy. (**C**) Comparison of growth conditions between CG-pp1 and LPZ.

To effectively track the *in vivo* kinetics route of LPZ, we utilized the cyan-green anaerobic fluorescent protein pp1 (20–22). We engineered a plasmid suitable for the expression of this protein in LPZ and successfully generated the cyan-green LPZ-pp1 (CG-pp1) strain via electroporation (Fig. 1A). We then assessed the phenotypic differences between LPZ and CG-pp1. Microscopic and colony morphology analyses revealed that CG-pp1 retained the characteristic round, white colonies, Gram-positive staining, and short rod-shaped morphology of the original strain. Furthermore, growth curve analysis demonstrated no significant difference in growth kinetics between CG-pp1 and LPZ (Fig. 1B and C). These findings indicate that the introduction of the fluorescent label did not alter the biological characteristics of LPZ, confirming the suitability of CG-pp1 for *in vivo* kinetic studies.

#### *In vivo* kinetics of liquid CG-pp1 in mice

**Fig 2:**
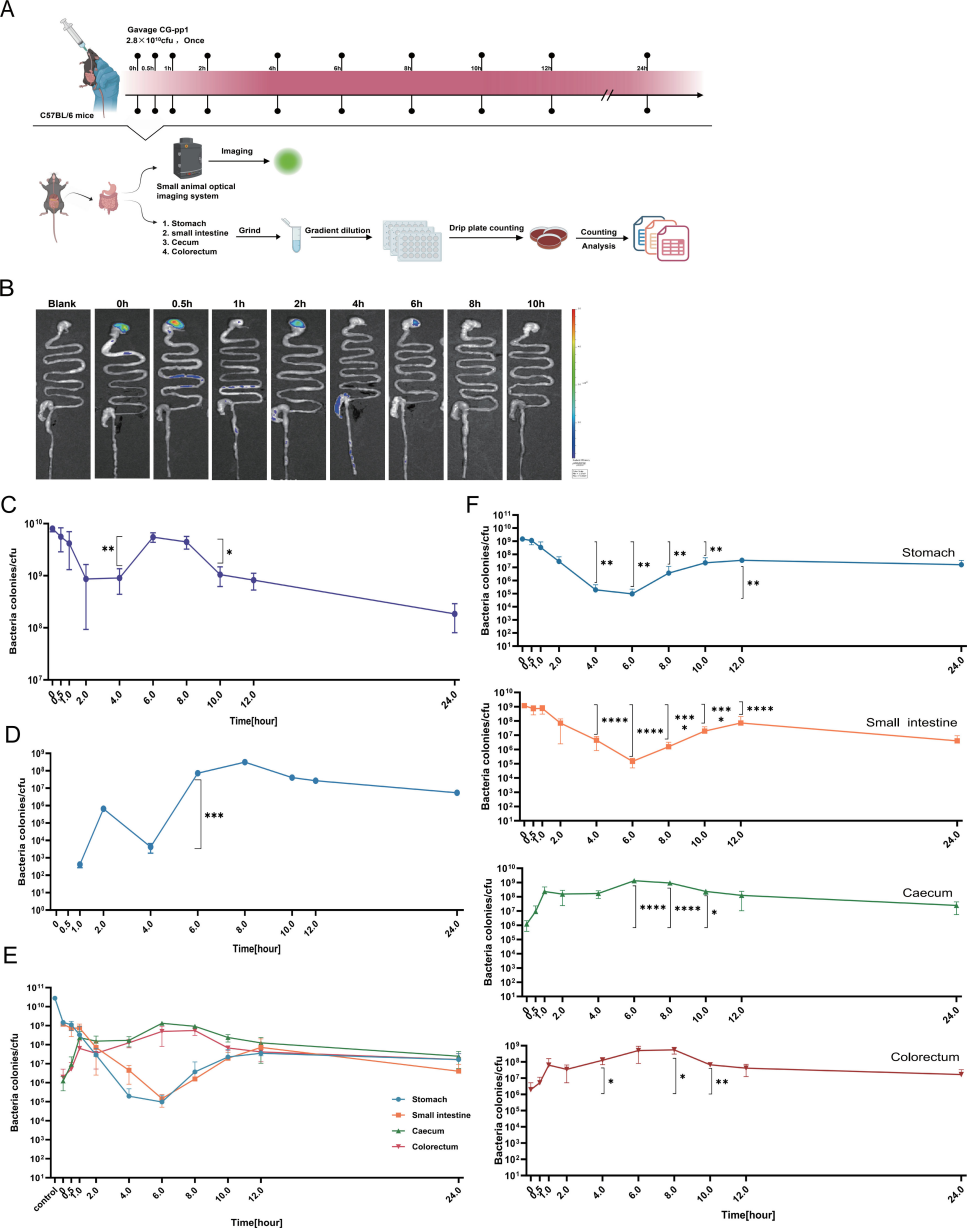
The kinetics of liquid CG-pp1 *in vivo*. (**A**) Study design and flow diagram. The kinetic time points were set at 0, 0.5, 1, 2, 4, 6, 8, 10, 12, and 24 h. The movement trajectory of CG-pp1 in the animals was observed using a small animal optical imaging system, and CG-pp1 loads in different tissues at various time points were quantified by the drop plate method. This study aimed to characterize the *in vivo* kinetics of liquid CG-pp1, determining its dynamic profiles, migration routes, and persistence across tissues and time points post-ingestion. (**B**) Continuous monitoring of liquid CG-pp1 *in vivo* using a small animal optical imaging system until the fluorescence signal disappeared. *n* = 4. (**C**) Temporal changes in liquid CG-pp1 count throughout the gastrointestinal tract tissues. (**D**) Temporal changes in liquid CG-pp1 counts in excreted fecal samples over time. (**E**) Quantification of liquid CG-pp1 in diverse *in vivo* locations at different time points following ingestion. *n* = 4. (**F**) Line graph showing dynamic changes in liquid CG-pp1 counts in four tissue groups (stomach, small intestine, cecum, and colorectum) within 24 h. Comparisons among groups were based on differences relative to 0 h. Additionally, in the stomach group, a specific comparison was made between 12 h and the lowest point at 6 h. Data are presented as mean ± SEM. Statistical significance was determined using two-tailed student’s *t*-test or two-way ANOVA. Significance levels: *P < 0.05, **P < 0.01, ***P < 0.001, and ****P < 0.0001.

Utilizing the CG-pp1 strain, we characterized its *in vivo* kinetics in mice maintained on an alfalfa-free diet. We initially tracked the temporal localization of liquid CG-pp1 within the gastrointestinal tract. By harvesting the intestines at predetermined intervals and employing fluorescence imaging, we mapped the transit of 2.8 × 10^10^ CFU CG-pp1 through the stomach, small intestine, cecum, and colorectum. These imaging data were corroborated by quantifying viable CG-pp1 counts from each intestinal segment at matching time points (Fig. 2A). Distinct CG-pp1 boluses were observed in the stomach and small intestine immediately post-ingestion. Notably, CG-pp1 transited the small intestine within the first hour and reached the cecum (Fig. 2B), where they retained for several hours before gradually progressing to the colorectum.

Additionally, at the initial time point, approximately 28.57% of the ingested CG-pp1 were detected within the gastrointestinal tract (Fig. 2C). The CG-pp1 load subsequently experienced a sharp decline during the 0–2 h interval, a trajectory consistent with the early fecal excretion patterns (Fig. 2D). Longitudinal analysis demonstrated significant CG-pp1 proliferation at 4–8 h post-ingestion relative to the 0 h baseline (Fig. 2C, E and F). This proliferative phase aligned with the observed dynamics in fecal excretion (Fig. 2D), suggesting that CG-pp1 enters a peak reproductive phase during the 4–8 h window. Notably, these findings were corroborated by trends in the cecum and colorectum (Fig. 2F), implying that these specific regions serve as the primary niches for CG-pp1 replication. Furthermore, correlation analysis identified the strongest positive correlation in the colorectum (*R* = 0.949, *P* = 0.001), followed by the cecum (*R* = 0.846, *P* = 0.017). In contrast, the small intestine (*R* = −0.802, *P* = 0.03) and stomach (*R* = −0.722, *P* = 0.067) exhibited negative correlations. These data robustly support the specific association of CG-pp1 with the colorectum. Finally, the observed reduction in CG-pp1 abundance within the gastrointestinal tract after 8 h may be attributed to competitive exclusion from ecological niches.

#### *In vivo* kinetics of lyophilized powder CG-pp1 in mice

**Fig 3:**
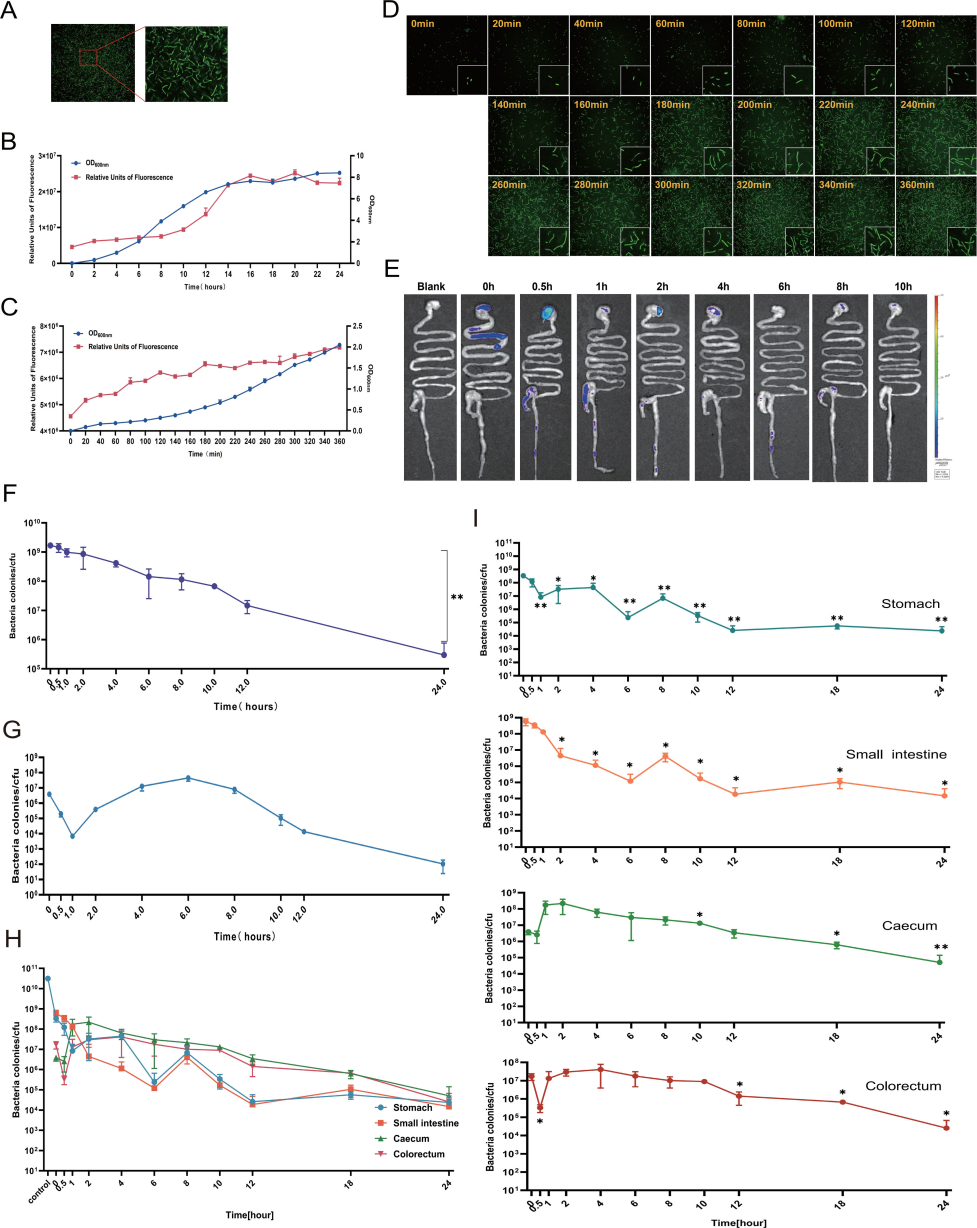
The kinetics of Lyophilized Powder CG-pp1 *in vivo*. (**A**) Verification of *in vitro* fluorescence efficacy of lyophilized powder CG-pp1. (**B**) Growth characteristics and associated fluorescence changes of lyophilized powder CG-pp1. (**C**) *In vitro* resuscitation growth and concomitant fluorescence changes of lyophilized powder CG-pp1 (monitored within 6 h). (D) *In vitro* morphological observation of resuscitated lyophilized powder CG-pp1 (observed at 6 h). (**E**) Continuous monitoring of lyophilized powder CG-pp1 *in vivo* using a small animal optical imaging system until the fluorescence signal disappeared. *n* = 4. (**F**) Continuous observation of changes in lyophilized powder CG-pp1 counts in the entire gastrointestinal tract tissue over time. (**G**) Temporal changes in lyophilized powder CG-pp1 counts in excreted fecal samples over time. (**H**) Quantification of lyophilized powder CG-pp1 in various *in vivo* locations at different time points post-ingestion. *n* = 4. (**I**) Line graph showing dynamic changes in lyophilized powder CG-pp1 counts across four tissue groups (stomach, small intestine, cecum, and colorectum) within 24 h. Comparisons among groups were based on differences relative to 0 h. Data are presented as mean ± SEM. Statistical significance was determined using two-tailed student’s *t*-test or two-way ANOVA. *P < 0.05 and **P < 0.01.

We sought to determine whether lyophilized powder CG-pp1 displays *in vivo* kinetic characteristics comparable to, or exceeding, those of the liquid formulation. We initially verified the retention of cyan-green fluorescence in the lyophilized powder CG-pp1 preparation. As anticipated, the lyophilized powder CG-pp1 exhibited robust fluorescence intensity at 488/514 nm, with absolute values comparable to those of the liquid CG-pp1 formulation at their respective optimal excitation and emission wavelengths (Fig. 3A). To further characterize the growth status post-lyophilization, we correlated *in vitro* growth curves with fluorescence monitoring (Fig. 3B and C). The data revealed a positive correlation between fluorescence intensity and CG-pp1 growth, with the population reaching the stationary phase by the 16th hour (Fig. 3B).

A key focus of our investigation was the resuscitation kinetics of lyophilized powder CG-pp1 transitioning from a dormant state to active metabolism. We monitored morphological changes in lyophilized powder CG-pp1 over a 6-h period (Fig. 3C). Initial elongation was observed at 40 min. Between 60 and 80 min, significant elongation and unfolding occurred, resulting in markedly elongated cells. By 100 to 120 min, the CG-pp1 cells had fully extended and unfolded, initiating cellular replication (Fig. 3D). For the *in vivo* assessment, mice were gavaged with 2.8 × 10¹^0^ CFU of lyophilized powder CG-pp1, matching the CG-pp1 count of the liquid formulation. We subsequently monitored fluorescence intensity changes at various time points as the CG-pp1 traversed the stomach, small intestine, cecum, and colorectum (Fig. 3E). Concurrently, we precisely quantified variations in CFU counts across different time intervals and anatomical locations (Fig. 3F).

Approximately 6.01% of the ingested CG-pp1 were detected at the 0-h time point (Fig. 3F). As the experiment progressed, the total CG-pp1 load within the gastrointestinal tract demonstrated a gradual decline. Concomitantly, the fecal CG-pp1 count, which was initially elevated, also decreased progressively (Fig. 3G). Notably, there was no evidence of CG-pp1 proliferation or active growth at any point during the experimental period (Fig. 3H and I). We further observed that the lyophilized powder CG-pp1 was rapidly translocated from the stomach and small intestine to the cecum and colorectum within 30 min. Fluorescent CG-pp1 was concurrently detected in the stomach and colorectum (Fig. S1A and D). The CG-pp1 load in the cecum peaked at 2nd hour (2.19 × 10^8^ CFU) before gradually declining, coinciding with the continuous excretion of CG-pp1 from the colorectum. Specifically, the colorectum harbored 1.32 × 10^7^ CFU at 10th hour and 5.0 × 10^4^ CFU at 24th hour. Crucially, a marked disparity in CG-pp1 retention was evident between the liquid and lyophilized powder formulations. At 24th hour post-ingestion, the retention rate of the liquid formulation was 270- to 680-fold higher than that of the lyophilized powder (Table 1). Taken together, these findings indicate that while the *in vivo* kinetic profiles of different formulations share general similarities, significant differences exist.

**TABLE 1:** Comparison of retention between two formulations CG-pp1 in different tissues at 24th hour post-ingestion

Tissue	Liquid CG-pp1 (CFU)	Lyophilized powder CG-pp1 (CFU)	Ratio (liquid CG-pp1/lyophilized powder CG-pp1)
Stomach	1.63 × 10^7^	2.38 × 10^4^	680
Small intestine	4.04 × 10^6^	1.49 × 10^4^	271
Caecum	2.46 × 10^7^	5.0 × 10^4^	490
Colorectum	1.66 × 10^7^	2.5 × 10^4^	664

### Analyzing CG-pp1-mediated regulation of the gut microbiota-metabolite axis

#### Metagenomic alterations in the Z8h group relative to the negative group

Integrating the kinetic data from both formulations, liquid CG-pp1 demonstrated the ability to proliferate within the gastrointestinal tract within 24 h. Notably, it retained a substantially higher CG-pp1 load (2.306%) at the 24th hour compared to the lyophilized powder CG-pp1 (0.017%). This superior transit efficiency positions the liquid formulation to more effectively exert its probiotic functions. Therefore, to investigate the impact of liquid CG-pp1 gavage on the intestinal microecology, we selected the 8th hour time point for metagenomic sequencing of cecal and colorectal contents (Z8h group), which corresponds to the peak proliferation phase observed *in vivo*.

**Fig 4:**
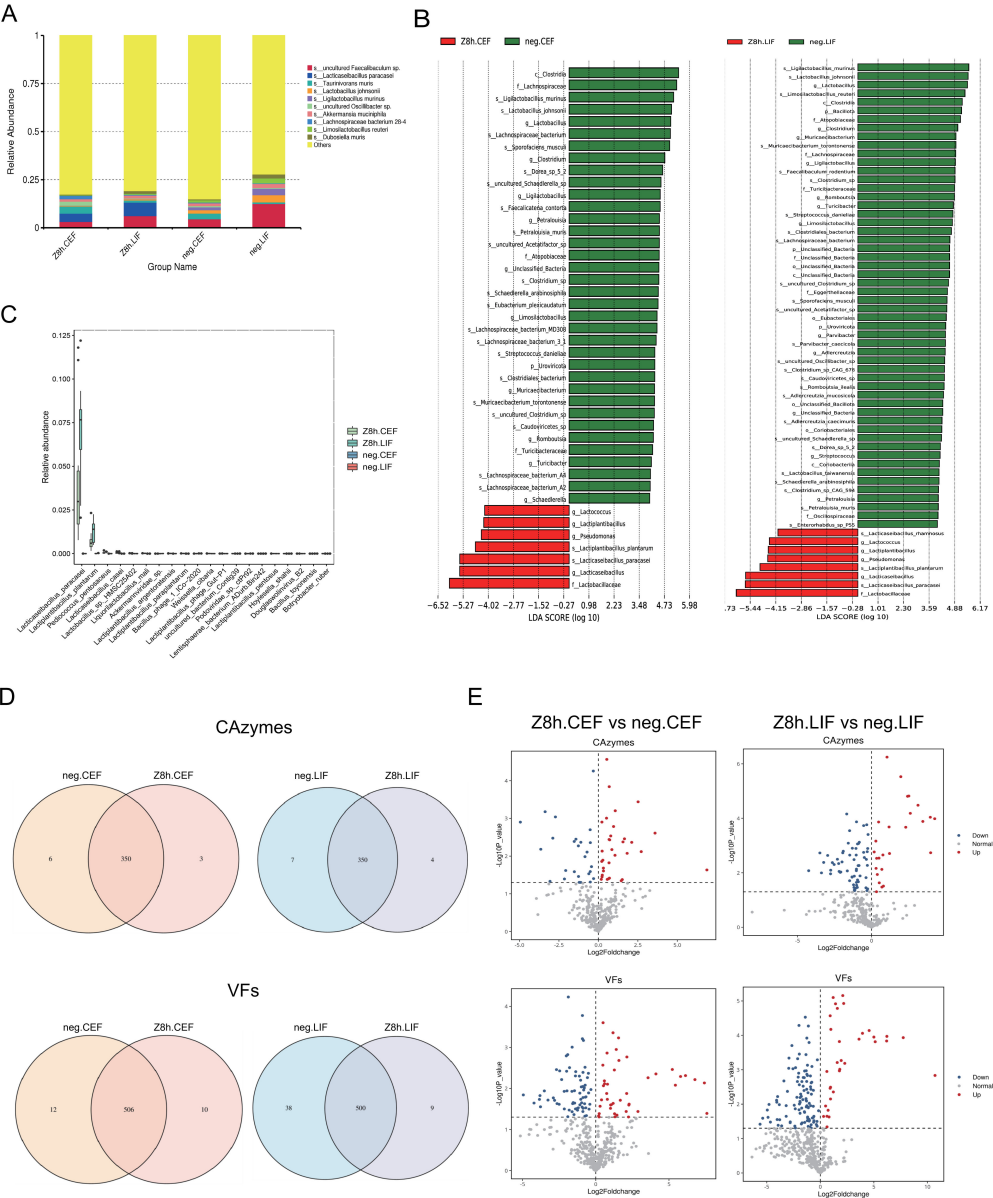
Taxonomic signatures of the gut microbiota in the Z8h and negative groups based on metagenomic sequencing. Z8h, group at 8 h after gavage with CG-pp1; Z8h.CEF, cecal feces sample from Z8h, *n* = 8; Z8h.LIF, colorectal feces sample from Z8h, *n* = 8; .neg, negative group; neg.CEF, cecal feces sample from neg, *n* = 9; neg.LIF, colorectal feces sample from neg, *n* = 10; LDA, Linear Discriminant Analysis. (**A**) The top 10 species with the highest maximum relative abundance in each group are shown; all remaining species are categorized as “Others.” (**B**) LEfSe analysis was performed to identify biomarkers with statistically significant differences between groups. The bar length represents the LDA score, indicating the magnitude of the impact of each differential species. Red and green nodes denote microbial groups that play a significant role in the red and green groups, respectively. (**C**) Abundances of the top 20 differential microorganisms between groups. (**D**) Number of shared and unique significantly altered functional genes (CAZymes and VFs) in CEF and LIF tissues from the neg and Z8h groups. (**E**) Comparison of significantly altered functional genes in CEF and LIF tissues between the neg and Z8h groups. A log2(Fold Change) > 0 indicates significant upregulation following 8 h of CG-pp1 intervention, while log2(Fold Change) < 0 indicates significant downregulation. Points closer to the top of the plot correspond to smaller *P* values, while points farther to the left or right indicate larger abundance changes.

Metagenomic profiling elucidated that, relative to the negative group, following CG-pp1 ingestion for 8 h markedly boosted the abundance of *L. paracasei* by 14,293-fold in the cecum and 23,211-fold in the colorectum. This magnitude of increase coincides with the kinetic trends at the species level (Fig. 4A; Table 2). LEfSe analysis further revealed a significant enrichment of beneficial taxa within the cecal and colorectal environments of the Z8h group. Key enriched genera/species included s_*Lactiplantibacillus plantarum*, s_*Lacticaseibacillus rhamnosus*, g_*Lacticaseibacillus*, g_*Lactiplantibacillus,* and g_*Lactococcus* (Fig. 4B). Most notably, *Lactobacillus plantarum* exhibited a substantial surge in abundance (Fig. 4C). Collectively, these findings indicate that at 8th hour post-ingestion, CG-pp1 effectively reshapes the microbial architecture and significantly promotes the proliferation of beneficial bacterial communities.

**TABLE 2:** Overview of the relative abundances of the top 10 species

Species name	Z8h.CEF	neg.CEF	Z8h.LIF	neg.LIF
s__uncultured *Faecalibaculum* sp.	3.1460%	4.5791%	6.2212%	12.5286%
s__*Lacticaseibacillus paracasei*	4.2880%	0.0003%	6.9634%	0.0003%
s__*Taurinivorans muris*	3.7187%	2.9033%	1.0959%	0.7823%
s__*Lactobacillus johnsonii*	0.3775%	1.7837%	0.6727%	3.7648%
s__*Ligilactobacillus murinus*	0.0727%	1.5219%	0.1704%	3.3660%
s__uncultured *Oscillibacter* sp.	2.0511%	0.6409%	0.2158%	0.3742%
s__*Akkermansia muciniphila*	1.3590%	1.3793%	1.5287%	2.1452%
s__*Lachnospiraceae* bacterium 28-4	1.7502%	0.4393%	0.3959%	0.2176%
s__*Limosilactobacillus reuteri*	0.1984%	1.1523%	0.4947%	2.5347%
s__*Dubosiella muris*	0.3801%	0.5227%	1.4459%	2.1332%
Others	82.6583%	85.0772%	80.7954%	72.1531%

Further analysis highlighted significant disparities in the distribution of carbohydrate-active enzymes (CAZymes) and virulence factors (VFs) between the negative and Z8h group (Fig. 4D and E). Notably, while significantly altered VFs in the Z8h group were predominantly downregulated, reflecting a decrease in pathogenic potential, the altered CAZymes were primarily upregulated. Specifically, the VFs that were significantly reduced are involved in various pathogenic mechanisms, including hemolysins, capsular polysaccharides, and secretion systems (T6SS and T3SS). In contrast, the enriched CAZyme genes predominantly encompassed glycoside hydrolases (GH), polysaccharide lyases (PL), and carbohydrate-binding modules (CBM), which are instrumental in plant fiber degradation, carbohydrate metabolism, and microbial growth. Consequently, CG-pp1 ingestion optimized the functional profile of the intestinal microbiota and mitigated the abundance of potential pathogenic genes.

#### Untargeted metabolomics profiles of the Z8h and negative group

**Fig 5:**
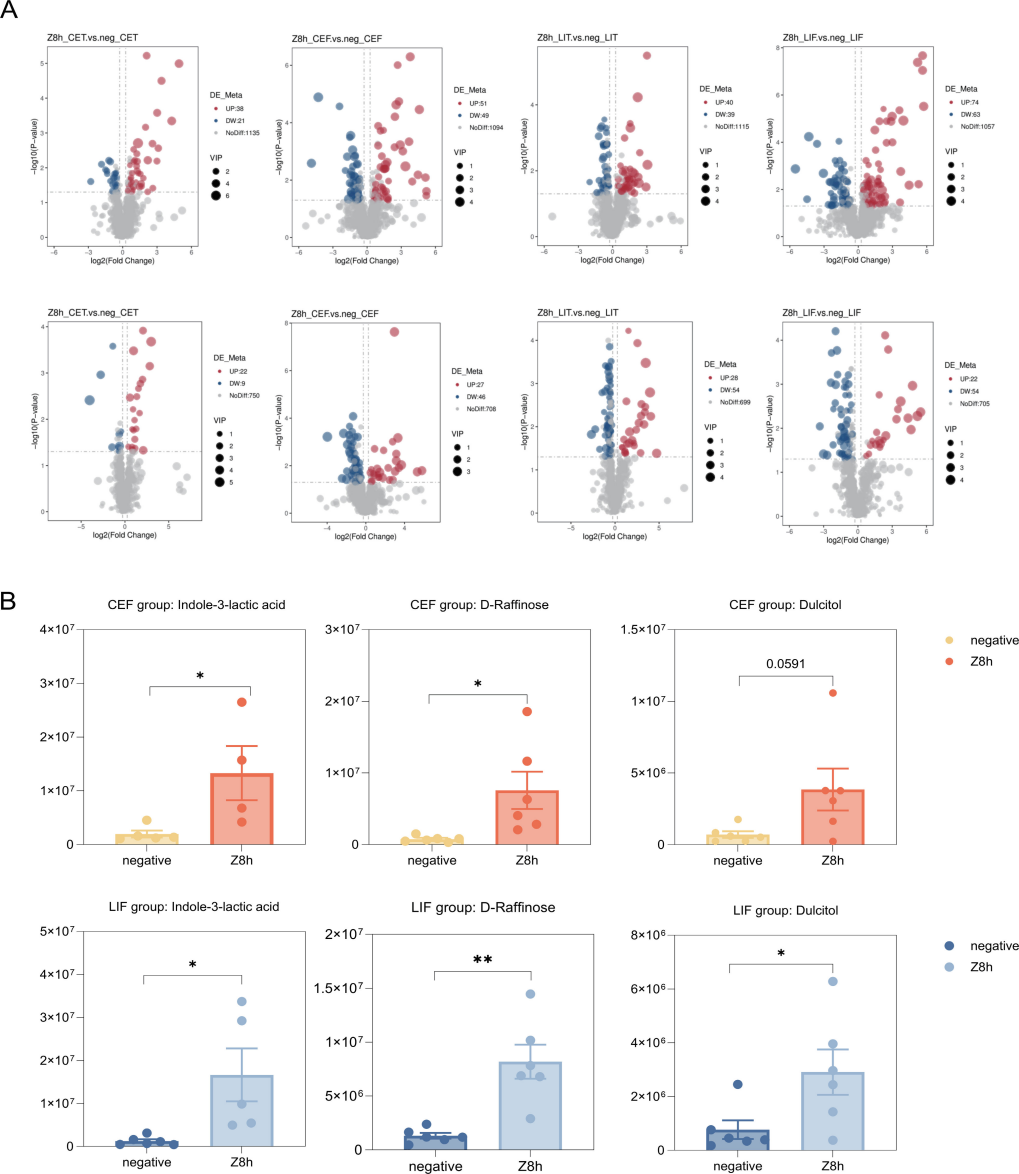
Metabolome profiles in the Z8h and negative groups. Z8h, group at 8 h after gavage with CG-pp1; Z8h_CET, cecal tissue sample from Z8h; Z8h_CEF, cecal fecal sample from Z8h; Z8h_LIT, colorectal tissue sample from Z8h; Z8h_LIF, colorectal fecal sample from Z8h; neg, negative group; neg_CET, cecal tissue sample from neg; neg_CEF, cecal fecal sample from neg; neg_LIT, colorectal tissue sample from neg; neg_LIF, colorectal fecal sample from neg. *n* = 6. (**A**) Volcano plots illustrating metabolite changes between the Z8h and neg groups in cecum tissue, colorectum tissue, and their respective feces. The *x*-axis indicates the log2-transformed fold change in fecal metabolite abundances, and the *y*-axis denotes the log10-transformed *Q* value. Horizontal lines represent the significance threshold (*Q* < 0.05). Red dots indicate significantly upregulated metabolites, while blue dots indicate significantly downregulated metabolites. (**B**) Boxplots showing representative metabolites that were significantly altered in CEF and LIF. Data are presented as mean ± SEM. Statistical significance was determined using two-tailed student’s *t*-test or two-way ANOVA. *P < 0.05 and **P < 0.01.

To investigate the intricate interplay between gut microbiota and host co-metabolism, we employed untargeted metabolomics on feces and intestinal tissues to characterize the metabolic changes induced by CG-pp1. Rigorous quality control was confirmed by the high correlation among QC samples (Pearson’s *r* = 1; Fig. S2A) and their tight clustering in Principal Component Analysis (PCA), which validated the stability of the analytical method (Fig. S2B). Partial least squares discriminant analysis (PLS-DA) subsequently revealed distinct metabolite profiles separating the Z8h group from the negative group across different tissues (Fig. S2C). Differentially abundant metabolites (*P*< 0.05, VIP > 1) were further screened via volcano plots. In tissue samples, positive ion mode identified 59 altered metabolites in the Z8h_CET vs neg_CET comparison (38 up, 21 down) and 79 in the Z8h_LIT vs neg_LIT comparison (40 up, 39 down). Negative ion mode identified 31 metabolites in the Z8h_CET vs neg_CET group (22 up, 9 down) and 82 in the Z8h_LIT vs neg_LIT group (28 up, 54 down). In fecal samples, the Z8h_CEF vs neg_CEF comparison yielded 100 metabolites in positive ion mode (51 up, 49 down) and 73 in negative ion mode (27 up, 46 down). Similarly, the Z8h_LIF vs neg_LIF comparison yielded 137 metabolites in positive ion mode (74 up, 63 down) and 76 in negative ion mode (22 up, 54 down) (Fig. 5A; Tables 3 and 4).

**TABLE 3:** Differential metabolites identified in positive ion mode in the 8 h post-ingestion^*a*^

	Z8h_CET vs neg_CET	Z8h_CEF vs neg_CEF	Z8h_LIT vs neg_LIT	Z8h_LIF vs neg_LIF
Up	38	51	40	74
Down	21	49	39	63
NoDiff	1,135	1,094	1,115	1,057

^*a*^ CG-pp1 group compared to the negative group.

**TABLE 4:** Differential metabolites identified in negative ion mode in the 8 h post-ingestion^*a*^

	Z8h_CET vs neg_CET	Z8h_CEF vs neg_CEF	Z8h_LIT vs neg_LIT	Z8h_LIF vs neg_LIF
Up	22	27	28	22
Down	9	46	54	54
NoDiff	750	708	699	705

^*a*^ CG-pp1 group compared to the negative group.

We then examined the top 20 annotated differential metabolites across the four comparison groups. Notably, indole-3-lactic acid, D-raffinose, and dulcitol exhibited significant increases in the Z8h_CEF and Z8h_LIF groups relative to the negative group. Indole-3-lactic acid, an indole derivative derived from microbial tryptophan metabolism, bolsters the intestinal mucosal barrier and promotes the colonization of beneficial genera such as *Lactobacillus* and *Bifidobacterium*, thereby maintaining microbial homeostasis. D-Raffinose, a trisaccharide, facilitates the proliferation of beneficial bacteria like *Lactobacillus* while suppressing pathogenic bacteria, contributing to a healthy gut environment. Meanwhile, dulcitol serves as an environment-dependent carbon source that is preferentially metabolized when the niche is colonized by specific strains. Following 8 h of CG-pp1 ingestion, the abundance of these three metabolites was significantly elevated in both the cecum (CEF) and colorectum (LIF) compared to the negative group. These findings underscore the potential of CG-pp1 intake to facilitate the niche colonization of *Lactobacillus*.

**Fig 6:**
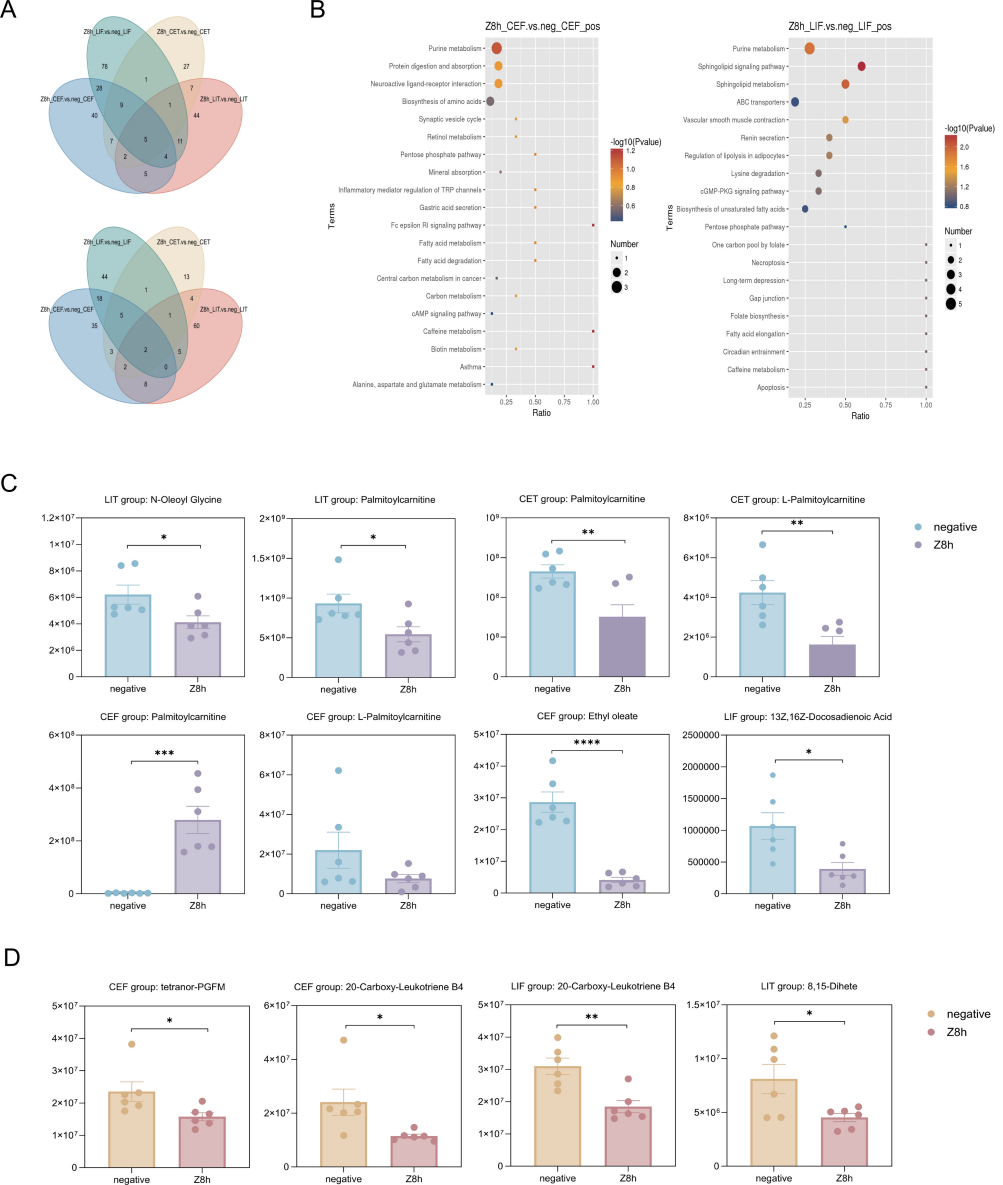
Taxonomic signatures and differential metabolic pathways. (**A**) Venn diagrams outlining the distinct taxonomic signatures associated with each group. (**B**) Results of metabolic pathway enrichment analysis for differential metabolites. Pathways with a *P* < 0.05 were considered significantly enriched. The *x*-axis represents the Rich Factor; a higher value indicates a greater ratio of differential metabolites annotated to that pathway. Dot size reflects the number of differential metabolites associated with the pathway. Enrichment analysis was based on metabolites annotated in the KEGG database. (**C**) Differential intestinal metabolites related to lipid metabolism. (**D**) Differential intestinal metabolites related to inflammatory mediators. Significance levels: *P < 0.05, **P < 0.01, ***P < 0.001, and ****P < 0.0001.

Venn diagram analysis revealed a total of 269 metabolites in positive ion mode and 201 in negative ion mode (Fig. 6A). Comparison of sample types showed that the colorectum harbored a greater number of differential metabolites than the cecum, reflecting a more diverse metabolic profile in the colorectum (Table 5). Clustering analysis demonstrated consistent patterns among samples of the same type though distinct differences in metabolite profiles were evident in the Z8h group (Fig. S3). We further investigated the results of metabolic pathway enrichment analysis. Bubble plot visualization indicated that purine metabolism was the most significantly altered pathway in positive ion mode for both comparisons (Z8h_CEF vs neg_CEF and Z8h_LIF vs neg_LIF) (Fig. 6B). KEGG pathway mapping further revealed a significant decrease in xanthine levels (*P* < 0.05) in both groups (Fig. S4). Given that xanthine is an upstream intermediate in uric acid synthesis, this reduction implies that CG-pp1 ingestion may inhibit the production of downstream uric acid. This finding aligns with previous research by Heping Zhang of Inner Mongolia Agricultural University, who demonstrated that the LPZ-containing probiotic complex Probio-X significantly lowers serum uric acid levels (23), thereby validating the hypouricemic potential of CG-pp1. Concurrently, CG-pp1 ingestion significantly attenuated intestinal metabolites associated with lipid metabolism (Fig. 6C). Notably, palmitoylcarnitine, an intermediate in mitochondrial fatty acid β-oxidation, was markedly decreased. Since elevated palmitoylcarnitine is indicative of impaired fat oxidation and lipid accumulation, its reduction suggests that LPZ enhances oxidative efficiency and mitigates lipid buildup. This supports the lipid-lowering potential of LPZ and is consistent with prior studies (24, 25). Similarly, other functionally related metabolites, including L-palmitoylcarnitine, N-oleoyl glycine, ethyl oleate, and 13Z,16Z-docosadienoic acid, were significantly reduced by CG-pp1 ingestion (Fig. 6C). Furthermore, we observed a significant reduction in lipoxygenase-derived inflammatory mediators such as tetranor PGFM, 20-carboxy-leukotriene B4, and 8,15-DiHETE (Fig. 6D). These findings suggest that LPZ exerts anti-inflammatory effects (26, 27), potentially contributing to improved lipid metabolism and overall metabolic health.

**TABLE 5:** Differential metabolites unique to each comparison group

	Z8h_CET vs neg_CET	Z8h_CEF vs neg_CEF	Z8h_LIT vs neg_LIT	Z8h_LIF vs neg_LIF
pos	27	40	44	78
neg	13	35	60	44

#### Integrated analysis of metagenomics and untargeted metabolomics

**Fig 7:**
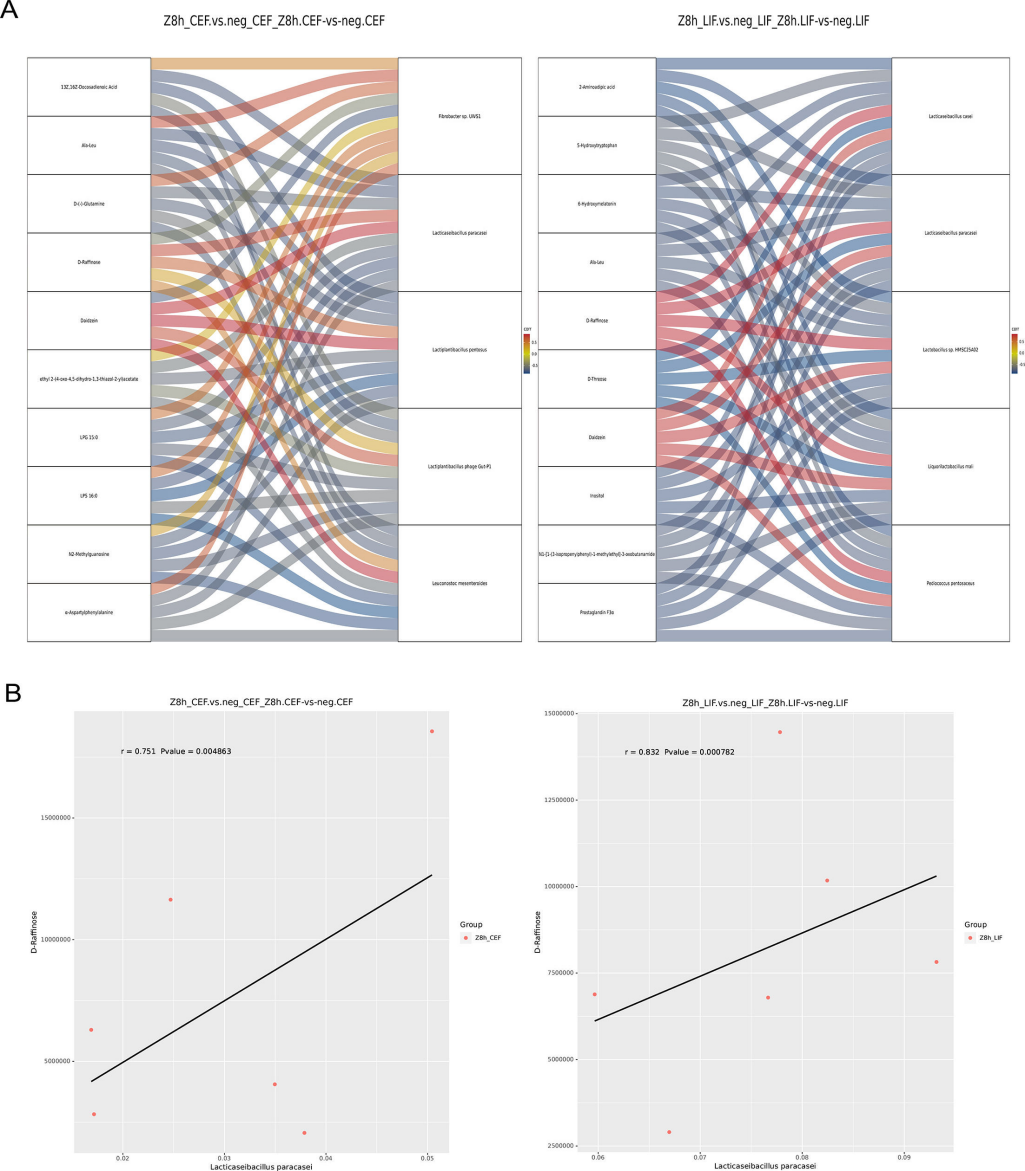
Microbial and metabolic associations of representative markers. (**A**) The Sankey diagram was constructed based on correlation coefficients between differential bacterial genera and differential metabolites. It illustrates the correlations between key metabolites and bacterial genera, reflecting the dynamic changes in these associations. (**B**) Correlation scatter plot showing the association between the specific metabolite D-raffinose and the microorganism *L. paracasei* across different groups, derived from the integration of metagenomics and untargeted metabolomics.

To elucidate the interplay between intestinal microorganisms and metabolites, we integrated metagenomic and untargeted metabolomic data for correlation analysis. A Sankey diagram demonstrated a robust correlation between L. paracasei and D-raffinose (*R* = 0.7511, *P* = 0.0048; Fig. 7A and B). As a functional oligosaccharide, D-raffinose promotes the growth of beneficial genera such as *Bifidobacterium* and *Lactobacillus* while suppressing pathogenic bacteria. This mechanism aligns with the observed *in vivo* increase in probiotic diversity, suggesting that LPZ ingestion cultivates a favorable intestinal ecosystem by enhancing both probiotic abundance and functional oligosaccharide levels. Notably, the increased abundance of *L*. paracasei was accompanied by elevated endogenous indole-3-lactic acid (Tables 6 and 7). Correlation analysis revealed a significant positive association between indole-3-lactic acid and *L. paracasei* in the cecum, validated in both tissue (*P* < 0.001) and fecal samples (*P* < 0.05). These findings imply that the cecum serves as an active site for indole-3-lactic acid synthesis, likely driven by the *L. paracasei*-induced remodeling of the intestinal microenvironment.

**TABLE 6:** Statistical correlations between the top 20 differential metabolites and *L. paracasei* in the Z8h_CET vs neg_CET and Z8h_CEF vs neg_CEF comparisons

Num	Metabolite	OTU	corr	*P* value
1	Indole-3-lactic acid	*L. paracasei*	0.938025331	0.00000649
2	D(+)-Phenyllactic acid	*L. paracasei*	0.906968969	0.0000469
3	Phenylacetaldehyde	*L. paracasei*	0.90312364	0.000057
4	trans-Cinnamic acid	*L. paracasei*	0.888786479	0.000110864
5	β-Gluconic acid β-lactone	*L. paracasei*	0.856767997	0.000371412
6	Myricetin 3-O-beta-D-galactopyranoside	*L. paracasei*	0.814166517	0.001265,892
7	Melatonin	*L. paracasei*	0.8054434	0.001567491
8	3-Hydroxybutyric acid	*L. paracasei*	−0.752081577	0.004779548
9	Daidzein	*L. paracasei*	0.746200656	0.005316134
10	Milbemectin A3	*L. paracasei*	0.741613365	0.005765277
11	5-Phenyl-N-(4-(trifluoromethyl)phenyl)oxazol-2-amine	*L. paracasei*	0.72801568	0.007265404
12	N1-[4-(aminosulfonyl)phenyl]−2,2-dimethylpropanamide	*L. paracasei*	0.667737721	0.017650326
13	16-Hydroxyhexadecanoic acid	*L. paracasei*	−0.660447179	0.019402191
14	Aldosterone	*L. paracasei*	0.65375638	0.021118821
15	4-[4-(tert-butoxycarbonyl)piperazino]−4-oxobut-2-enoic acid	*L. paracasei*	0.640021922	0.02498578
16	Estriol	*L. paracasei*	−0.637940677	0.02561361
17	L-(+)-Tartaric acid	*L. paracasei*	0.626315791	0.029332298
18	Ethyl 2-(4-oxo-4,5-dihydro-1,3-thiazol-2-yl)acetate	*L. paracasei*	−0.597499145	0.04020514
19	LPG 18:2	*L. paracasei*	0.595187231	0.04118605
20	β-Aspartylphenylalanine	*L. paracasei*	−0.59238207	0.042398898

**TABLE 7:** Statistical correlations between the top 20 differential metabolites and *L. paracasei* in the Z8h_CEF vs neg_CEF comparison

Num	Metabolite	OTU	corr	*P* value
1	LPG 19:1	*L. paracasei*	0.854188421	0.000404241
2	Daidzein	*L. paracasei*	0.852951741	0.000420758
3	LPG 18:3	*L. paracasei*	0.823344402	0.000998951
4	3-[(methoxycarbonyl)amino]−2,2,3-trimethylbutanoic acid	*L. paracasei*	0.814679387	0.001249668
5	(2R)−2,3-Dihydroxypropanoic acid	*L. paracasei*	0.804262585	0.001612211
6	Orotidine	*L. paracasei*	0.799853746	0.001787893
7	4-[4-(tert-butoxycarbonyl)piperazino]−4-oxobut-2-enoic acid	*L. paracasei*	0.763796841	0.003833535
8	D-Raffinose	*L. paracasei*	0.75113255	0.00486324
9	L-Threonine	*L. paracasei*	0.726560717	0.007441696
10	Royal jelly acid	*L. paracasei*	0.720298224	0.008237328
11	PC 16:0_20:4	*L. paracasei*	0.720298224	0.008237328
12	2-Hydroxycaproic acid	*L. paracasei*	0.683458312	0.014270856
13	(S)-Leucic acid	*L. paracasei*	0.676672329	0.015665024
14	ST 29:2;O;S	*L. paracasei*	0.67174688	0.016737937
15	Aldosterone	*L. paracasei*	0.653090537	0.021295509
16	Stachyose	*L. paracasei*	0.645520271	0.023380867
17	Indole-3-lactic acid	*L. paracasei*	0.640208601	0.024930017
18	N-Acetyl-L-carnosine	*L. paracasei*	0.638360826	0.025485957
19	Phenylacetaldehyde	*L. paracasei*	0.575902559	0.050039479
20	D(+)-Phenyllactic acid	*L. paracasei*	0.575739677	0.050119499

## DISCUSSION

Our findings demonstrate that high-activity liquid LPZ persists in the intestine for 24 h with a residual ratio of 1/664. This probiotic not only augments the diversity and abundance of beneficial commensals but also upregulates CAZymes, thereby optimizing gut microbiota metabolic function and enhancing host carbohydrate metabolism. Notably, the gastrointestinal kinetics of LPZ exhibit a marked formulation-dependent asymmetry. At 24th hour post-ingestion, the residual amount of high-activity liquid CG-pp1 is 270- to 680-fold greater than that of lyophilized powder CG-pp1, underscoring the superior short-term retention of the liquid formulation. This dramatic disparity is attributed to the synergistic interplay of physical protection, cellular physiology, and colonization strategies. The high-viability liquid formulation utilizes its native carrier to provide enhanced buffering and protection during upper gastrointestinal transit. Crucially, the metabolically active “pioneer” state of these liquid CG-pp1 enables an immediate response to gastrointestinal stressors, obviating the requirement for a delicate reactivation phase. Consequently, these primed liquid CG-pp1 possess a competitive advantage, rapidly initiating colonization mechanisms such as biofilm formation to establish effective short-term retention. In contrast, lyophilized powder CG-pp1, while advantageous for long-term storage, is constrained by a “dormancy-recovery” paradigm during the acute 24 h gut challenge. This leads to substantial CG-pp1 attrition during rehydration, accounting for the precipitous drop in residual counts. Utilizing advanced molecular imaging techniques, we precisely characterized the *in vivo* kinetic profiles of LPZ across different formulations, elucidating distinct transit efficiencies regarding distribution, migration, and metabolic activity.

Previous studies have employed near-infrared fluorescent protein IRFP713 to monitor the migration of *Lactobacillus plantarum* along the gastrointestinal tract, documenting the fluorescence signal decay kinetics of *Lactobacillus plantarum*-713 (28). Since LPZ lacks the necessary heme genes for near-infrared labeling (29, 30), we utilized the cyan-green anaerobic fluorescent protein pp1, which demonstrated a comparable signal decay profile. Although fluorescent probiotics enable real-time tracking of kinetic behavior, the use of plasmid-embedded vectors (31) presents a potential limitation. Plasmid instability during cell division can lead to signal attenuation, especially under antibiotic-free selective pressure, potentially preventing the visualization of the total LPZ population. To mitigate this, our study superimposed real-time CG-pp1 count statistics, thereby compensating for the decline in fluorescence imaging sensitivity for CG-pp1 after 6 h. Thus, our results provide a comprehensive characterization of the 24-h dynamic retention of LPZ in the gastrointestinal tract. As the precise time-varying trajectories of probiotics remain largely unclear, these findings provide new insights for the application of probiotics in modulating gut microbiota for preventive and therapeutic purposes.

Exogenous probiotic ingestion has been demonstrated to augment the abundance of beneficial species in the intestinal microbiota (32). Concordantly, our metagenomic sequencing revealed that LPZ ingestion effectively enriched probiotic species in the colorectal region, particularly s_*Lactiplantibacillus plantarum*, s_*Lacticaseibacillus rhamnosus*, g_*Lacticaseibacillus*, g_*Lactiplantibacillus,* and g_*Lactococcus*. This phenomenon demonstrates the significant cross-feeding effect and ecological niche remodeling mechanisms within the gut microbiota (33, 34). Specifically, acting as a pioneer strain, LPZ generates specific metabolic products (such as lactate, short-chain fatty acids, and oligosaccharides) that directly serve as utilizable substrates for other beneficial bacteria (e.g., *Lacticaseibacillus rhamnosus* and *Lactococcus*). This drives the synergistic proliferation of the entire probiotic community through a nutrient cascade effect. Concurrently, the substantial organic acids produced by LPZ fermentation significantly lower the local intestinal pH, creating an acidic environment that is unfavorable for the survival of pathogenic bacteria, thereby effectively inhibiting the growth of competitors. This competitive inhibition releases more nutritional resources and living space for the lactic acid bacteria group, thereby expanding their ecological niche and ultimately leading to a comprehensive increase in the abundance of beneficial species. Whereas microbiota analysis identifies “who is present,” metabolomics captures “what has been done,” underscoring the importance of metabolites in assessing system functionality (35). Consequently, we characterized the metabolic profile associated with intestinal LPZ colonization. Notably, LPZ ingestion significantly reduced xanthine levels in mouse intestinal contents. Given that xanthine is a direct precursor of uric acid (36), its dysregulation is implicated in gout and related pathologies (37, 38). Moreover, LPZ abundance in cecal and colorectal feces exhibited a strong positive correlation with daidzein (39, 40), a metabolite known to ameliorate dyslipidemia by reducing serum total cholesterol and low-density lipoprotein (LDL). Clinically, the LPZ-containing formulation Probio-X has been shown to lower serum uric acid in hyperuricemic patients, as well as reduce total cholesterol, triglycerides, and LDL levels (32). These findings substantiate the dual capacity of this probiotic to mitigate both uric acid and lipid levels. Furthermore, the LPZ-induced reduction in uric acid was accompanied by a proliferation of beneficial bacteria, such as *Lactobacillus plantarum*, relative to the negative group (32). We also identified a robust correlation between LPZ abundance and the prebiotic D-raffinose. D-Raffinose is selectively utilized by beneficial genera like *Bifidobacterium* and *Lactobacillus* to sustain microbial equilibrium (41–43). Furthermore, emerging evidence indicates that indole-3-lactic acid plays a pivotal role in promoting the growth and reproduction of beneficial gut bacteria, including *Bifidobacterium* and *Lactobacillus*, thereby contributing to the maintenance of a balanced intestinal microbiota. It is noteworthy that indole-3-lactic acid has been reported to exhibit anxiolytic properties, suggesting its potential as a neuromodulator acting through the gut-brain axis. Although this prospect is promising, definitive confirmation will require further behavioral research. This discovery significantly advances our understanding of the complex interplay between the gut microbiome and host physiology.

Informed by our kinetic research findings and derived from pharmacokinetic principles (44, 45), we propose the novel concept of “probiotikinetics.” Distinct from pharmacokinetics, probiotikinetics characterizes the *in vivo* dynamic processes of probiotics, encompassing colonization, proliferation, metabolism, and excretion. By synthesizing microbial ecology theories with kinetic modeling, this approach allows for the quantitative analysis of interactions between probiotics and the host intestinal microenvironment. Building on our results, the potential future applications of probiotikinetics include:

### Optimization of probiotic formulations

Kinetic models that predict colonization efficiency and compare liquid vs lyophilized powder formulations can guide the optimization of formulation design to enhance gastric acid tolerance. Moreover, by precisely defining the dose-response relationship through the kinetic chain of “viable count–colonization time–metabolite production,” optimal dosages can be established. This strategy obviates the drawbacks associated with traditional “blind high-dose supplementation.”

### Development of strategies for disease intervention

LPZ mediates xanthine reduction via purine metabolism regulation; kinetic models can predict the time-effect profile of this uric acid-lowering action, facilitating the design of gout treatment protocols. Furthermore, the anxiolytic properties of indole-3-lactic acid are intrinsically linked to probiotic metabolic kinetics. Modeling the “probiotic proliferation–metabolite production–neuroregulation” cascade enables the development of targeted interventions targeting the “gut-brain axis.”

### Theoretical framework for microecological regulation

Analyzing the interaction kinetics between probiotics and host metabolites provides a foundation for constructing combined “probiotic-prebiotic” intervention models (synbiotics).

Distinct from pharmacokinetics, the core *in vivo* processes of probiotics follow a colonization-proliferation-metabolism-excretion (CPME) cycle. Whereas small-molecule drugs typically exhibit monotonic decay over time, viable bacterial counts follow an S-shaped “proliferation-plateau-decay” trajectory. This kinetics are governed by the host intestinal microecology (competition with indigenous flora, mucus layer barriers), diet (dietary fiber, prebiotics), and the physiological environment (pH, oxygen partial pressure). The mechanisms of action are notably more complex: viable bacteria exert effects primarily through metabolites (e.g., short-chain fatty acids [16, 46] and indole derivatives [47, 48]), microbial interactions (inhibition of pathogens [49, 50] and promotion of beneficial taxa [51]), and host immune regulation (52). In terms of dose-response relationships, viable bacteria demonstrate a “threshold effect,” necessitating a minimum effective colonization load, with efficacy indirectly mediated by metabolite accumulation. While traditional pharmacokinetics posits a direct positive correlation between drug concentration and effect, probiotikinetics transcends this linear framework by integrating the dynamic interactions of microbial ecology. This perspective provides an interdisciplinary theoretical foundation for basic research and facilitates precise applications in disease prevention and functional food development. Notably, viable bacteria possessing high metabolic activity and strong adhesion capabilities can rapidly colonize and modulate the microecology, offering a rapid onset suitable for acute interventions. For instance, LPZ administered prior to a high-purine meal reaches a reproductive peak at 6–8 h post-ingestion, significantly influencing intestinal purine metabolism while enhancing prebiotic production and probiotic abundance. This highlights the potential of LPZ in targeted metabolic regulation.

The primary innovation of this study is the visualization and characterization of the kinetic profiles of CG-pp1 within the gastrointestinal tract across different formulations. Our results demonstrate that liquid LPZ exhibits enhanced *in vivo* proliferation and superior retention rates compared to lyophilized powder formulations. Notably, upon reaching peak proliferation, liquid LPZ not only stimulates the growth of commensal probiotics and metabolite production but also upregulates CAZymes while attenuating VFs, thereby amplifying the functional efficacy of LPZ. Concurrently, liquid LPZ significantly reduces *in vivo* xanthine levels, thereby attenuating the substrate availability for uric acid generation. Based on these findings, we propose the novel concept of “probiotikinetics.” These findings offer fresh insights into LPZ ingestion and provide a critical evidence base for its application in healthy populations. Further prospective studies are warranted to validate the long-term clinical effects and therapeutic potential of LPZ and related probiotic species.

## MATERIALS AND METHODS

### Bacterial strains, media, and culture conditions

The bacterial strains used in this study are listed in Table 8. The LPZ strain, isolated from traditional fermented mare’s milk, was provided by Heping Zhang. LPZ was cultured at 37°C in MRS media without aeration. LPZ-pp1 was cultured at 37°C in MRS media supplemented with 10 μg/mL chloramphenicol without aeration. Cultures were subsequently propagated in a Coy anaerobic chamber (5% H_2_, 20% CO_2_, balance N_2_) at 37°C.

**TABLE 8:** Bacterial strains and plasmids used in this study

	Relevant phenotype or genotype	Sourse or reference
Strains		
*E. coli* MC1061	Host microorganisms	Beijing Zoman Biotechnology Co., Ltd.
CECT925	*Lactobacillus reuteri*, reuterin-producing probiotic strain	Hangzhou Biosci Biotech Co., Ltd
LPZ	*L. paracasei*, probiotic strain	Inner Mongolia Agricultural University
CG-pp1	*L. paracasei*, probiotic strain	This study
Plasmid		
pNZ:BiEF1-pp1	pNZ8148 with promoter of elongation factor Tu of *Bifidobacterium longum* subsp. *infantis* ATCC 15697 replaced Pnis, insert the cyan-green anaerobic fluorescent protein pp1, Cmr	Proprietary to the laboratory
pNZ:LazTu-pp1	pNZ8148 with promoter of elongation factor Tu of *Lactobacillus reuteri* CECT925 replaced Pnis, insert the cyan-green anaerobic fluorescent protein pp1, Cmr	This study

### Construction of CG-pp1

#### Construction of the anaerobic fluorescent plasmid

The pNZ:BiEF1-pp1 vector was amplified by PCR using primers pNZ8148-pp1-F and pNZ8148-pp1-R to generate the pNZ8148-pp1 fragment. The promoter sequence was derived from the elongation factor Tu (*tuf*) gene of *Lactobacillus reuteri* CECT925 (ATCC 23272). This fragment was amplified using primers tu23272-F and tu23272-R. Subsequently, the PCR product was seamlessly cloned into the pNZ8148-pp1 vector. The ligation mixture was used to transform *E. coli* MC1061, and the resulting plasmid was designated pNZ:LazTu-pp1. All oligonucleotide sequences are listed in Table 9.

**TABLE 9:** Primers used in this study

Oligonucleotides	Sequence (5′−3′)	Description
pNZ8148-pp1-F	ATCAGGAGGTTTTCATTAATGATCAACGCAAAACTCCTGC	For the amplification of the vector pNZ8148
pNZ8148-pp1-R	CATTTGTAAATGTATTTGGATCTGGAGCTGTAATATAAAA	
tu23272-F	ATATTACAGCTCCAGATCCAAATACATTTACAAATGAACA	For the amplification of the insert fragment EF-Tu-Pp1
tu23272-R	AGTTTTGCGTTGATCATTAATGAAAACCTCCTGATAATTT	
pNZ:LazTu-pp1-JD-F	TAATGTCACTAACCTGCCCCGTT	For the identification of pNZ:LazTu-pp1
pNZ:LazTu-pp1-JD-R	CAGTAATTGCTTTATCAACTGCT	

#### Transformation of CG-pp1 with pNZ:LazTu-pp1

LPZ was inoculated into 5 mL of MRS broth and incubated anaerobically at 37°C. A 250 μL aliquot of culture (OD_600nm_ ≈ 0.8–1.0) was transferred to 5 mL of pre-warmed MRS medium containing 2% glycine. When the OD_600nm_ reached 0.2–0.3, ampicillin was added to a final concentration of 10 μg/L. At an OD_600nm_ of 0.4–0.5, the cells were harvested by centrifugation (8,000 × *g*, 5 min, 4°C) and washed three times with ice-cold electroporation buffer (0.5 M sucrose, 7 mM dipotassium hydrogen phosphate, and 1 mM magnesium chloride, pH 7.4). The cell pellet was finally resuspended in 50 μL of ice-cold buffer and kept on ice for 20 min prior to electroporation. Electroporation was performed using a Gene Pulser and Pulse Controller apparatus (Bio-Rad, Richmond, CA, USA) with 5 μL of plasmid DNA (0.3 ng/μL) in a 0.2 cm cuvette at settings of 25 μF, 200 Ω, and 0.75 kV. Following pulsing, the cells were immediately resuspended in 950 μL of MRS broth and recovered anaerobically at 37°C for 3 h. The cells were then plated on MRS agar supplemented with chloramphenicol (10 μg/mL) and incubated at 37°C for 2–3 days under anaerobic conditions.

#### Detection of fluorescent bacteria

Strains harboring plasmids were cultured overnight in MRS broth containing chloramphenicol (10 μg/mL). Strains lacking the pNZ:LazTu-pp1 plasmid served as negative controls. Bacterial fluorescence was quantified using a High Content Analysis System, with triplicate samples analyzed for each strain. The positive strains, which were validated by PCR primer specificity and capable of growing in chloramphenicol-supplemented (10 μg/mL) MRS broth with pp1 cyan-green fluorescence, were designated as CG-pp1.

### Phenotypic comparison between CG-pp1 and LPZ

#### Observation of morphology

Strains LPZ and CG-pp1 were cultured overnight in MRS broth; CG-pp1 medium was supplemented with chloramphenicol (10 μg/mL). Cells were then plated on respective MRS agar plates and incubated at 37°C for 24 h under anaerobic conditions. Morphological comparisons included colony assessment, Gram staining, and scanning electron microscopy analysis.

### Growth observation

LPZ was cultured overnight in MRS broth, while CG-pp1 was cultured in MRS broth containing chloramphenicol (10 μg/mL). Growth curves were monitored by measuring OD_600nm_ every 2 h for 24 h. Briefly, 200 μL aliquots was collected at each interval and analyzed using a spectrophotometer. Each assay was performed in at least three independent replicates.

### Accuracy verification of lyophilized powder CG-pp1

Based on a concentration of 2 × 10^11^ CFU/g for the lyophilized powder CG-pp1 and referencing the liquid inoculum standard, the lyophilized powder CG-pp1 was inoculated into 50 mL of MRS medium (containing chloramphenicol, 10 μg/mL) at a 1:100 ratio and anaerobically incubated at 37°C for 24 h. Subsequently, 100 μL of the culture was aliquoted into a PhenoPlate 96-well plate (Revivity, USA) to assess using a High Content Analysis System, and the strain was identified by mass spectrometry.

### Growth curve of lyophilized powder CG-pp1

The lyophilized powder CG-pp1(concentration: 2 × 10^11^ CFU/g) was inoculated into 50 mL of MRS medium (containing chloramphenicol, 10 μg/mL) at a 1:100 inoculum ratio and cultured anaerobically at 37°C. A 200 μL sample of culture was taken to measure OD_600 nm_ using a spectrophotometer every 2 h.

### *In vitro* recovery of lyophilized powder CG-pp1

The lyophilized powder CG-pp1 (concentration: 2 × 10^11^ CFU/g) was inoculated into 50 mL of MRS medium (containing chloramphenicol, 10 μg/mL) at a 1:100 inoculum ratio and cultured anaerobically at 37°C. Subsequently, 200 μL aliquots was sampled at 20-min intervals to measure OD_600nm_ using a spectrophotometer, assess morphological changes via a High Content Analysis System, and measure fluorescence signal variations using the SpectraMax I3 (Molecular Devices, USA).

### Kinetics of liquid CG-pp1 and lyophilized powder CG-pp1 in the isolated gastrointestinal tract

#### Animals

C57BL/6 mice were housed in the SPF barrier at the Experimental Animal Center of the Academy of Military Medical Sciences and performed in accordance with national guidelines and regulations. six-week-old female mice were used in all experiments, and housed in metabolic cages. All mice were housed under a 12 h light and 12 h dark cycle.

#### Bacterial ingestion

##### Liquid CG-pp1 ingestion

Each mouse in the experimental group was orally gavaged with 2.8 × 10^10^ CFU of liquid CG-pp1, which was resuspended in 200 μL of PBS.

##### Lyophilized powder CG-pp1 ingestion

1.4 g of lyophilized powder CG-pp1 (concentration: 2 × 10^11^ CFU/g) was dissolved in 2 mL of PBS. Each mouse was then orally gavaged 200 μL of the suspension.

##### Blank group

Mice in the blank group were gavaged with 200 µL of PBS as a control.

### Sample collection

Gastrointestinal tracts were isolated at various time points following oral gavage with liquid or lyophilized powder CG-pp1. After removal of fat and gland, *ex vivo* fluorescence imaging was performed to evaluate fluorescence intensity across different time points. Subsequently, the tissues with contents from stomach, small intestine, cecum, and colorectum were homogenized separately under cold conditions. Serial dilutions of the homogenates were prepared, and each dilution was plated by the drop method in triplicate on MRS agar plates supplemented with chloramphenicol (10 μg/mL) and incubated anaerobically at 37°C for 24 h. Finally, CFUs were enumerated to determine the viable CG-pp1 counts.

### Gut microbiome analysis

#### Sample collection and DNA extraction

Fresh fecal samples were collected using sterile disposable gloves. Defecation was induced by gentle abdominal pressure, and samples were immediately transferred to sterile centrifuge tubes and stored at −80°C. Two sample types were collected from each mouse: cecal contents and colorectal tissue. Z8h, group at 8 h after gavage with CG-pp1; Z8h.CEF, cecal feces sample from Z8h; Z8h.LIF, colorectal feces sample from Z8h; neg, negative group; neg.CEF, cecal feces sample from neg; neg.LIF, colorectal feces sample from neg. Genomic DNA was extracted using the CTAB method. Briefly, samples were suspended in CTAB lysis buffer (containing lysozyme) and incubated in a 65°C water bath with periodic inversion to ensure complete lysis. Following centrifugation, the supernatant was mixed with an equal volume of phenol:chloroform:isoamyl alcohol (25:24:1, pH 8.0) and centrifuged at 12,000 rpm for 10 min. The aqueous phase underwent a second extraction with chloroform:isoamyl alcohol (24:1). The DNA was precipitated with isopropanol at −20°C, and the pellet was washed twice with 75% ethanol. After removing residual ethanol by pipetting, the pellet was air-dried at room temperature and dissolved in ddH₂O. Residual RNA was eliminated by treatment with RNase A (37°C, 15 min). Finally, DNA concentration and purity were assessed using an Agilent 5400 system.

#### Data processing and analysis

Libraries were constructed using the ABclonal Rapid Plus DNA Lib Prep Kit (RK20208, ABclonal, China). DNA was fragmented to 350 bp (LE220R-plus, Covaris, USA) and sequenced on an Illumina NovaSeq X Plus (PE150, Illumina, San Diego, CA, USA). After removing low-quality reads (Bowtie2), clean data were assembled using MEGAHIT to generate scaftigs. Gene prediction and redundancy removal were performed to build a non-redundant gene catalog. Taxonomic annotation was conducted using the Micro_NR database, and functional annotation was performed against the Kyoto Encyclopedia of Genes and Genomes (KEGG), Carbohydrate-Active Enzymes (CAZy), and Virulence Factor Database (VFDB). Sample clustering and dimensionality reduction were assessed using PCA. Differential analysis of species and functions was performed using MetagenomeSeq and LEfSe. Correlations were analyzed using R (version 2.15.3), with significance set at *P* < 0.05.

### Untargeted metabolomics based on LC/MS

#### Sample collection and metabolite extraction

Samples were collected from two groups: negative group and Z8h (group at 8 h after gavage with CG-pp1) group. Each group consisted of six mice. Four sample types were collected from each mouse: cecal contents, cecal tissue, colorectal contents, and colorectal tissue, yielding a total of 48 samples for metabolomic analysis. Z8h, group at 8 h after gavage with CG-pp1; Z8h_CET, cecal tissue sample from Z8h; Z8h_CEF, cecal fecal sample from Z8h; Z8h_LIT, colorectal tissue sample from Z8h; Z8h_LIF, colorectal fecal sample from Z8h; neg, negative group; neg_CET, cecal tissue sample from neg; neg_CEF, cecal fecal sample from neg; neg_LIT, colorectal tissue sample from neg; neg_LIF, colorectal fecal sample from neg. All procedures were conducted in accordance with the standard operating protocols of Novogene Co., Ltd. (Beijing, China). Specifically, 100 mg of each sample was homogenized in liquid nitrogen and transferred to an EP tube. Subsequently, 500 μL of 80% methanol aqueous solution was added, followed by vortexing. The mixture was allowed to stand on ice for 5 min and then centrifuged at 15,000 × *g* for 20 min at 4°C. An aliquot of the supernatant was diluted with mass spectrometry-grade water to a final methanol concentration of 53% and centrifuged again at 15,000 × *g* for 20 min at 4°C. The resulting supernatant was injected into a Vanquish UHPLC system (Thermo Fisher, USA) coupled with a Q Exactive HF, Q Exactive HF-X, or Orbitrap Exploris 480 mass spectrometer (Thermo Fisher, USA) for LC-MS analysis.

#### Data processing and statistical analysis

Raw data files generated by UHPLC-MS/MS were processed using Compound Discoverer 3.1 (CD3.1, Thermo Fisher Scientific) to perform peak alignment, peak picking, and metabolite quantification. Metabolites were annotated by searching against the KEGG (https://www.genome.jp/kegg/pathway.html), HMDB (https://hmdb.ca/metabolites), and LIPID Maps (http://www.lipidmaps.org/) databases. For multivariate statistical analysis, data were transformed using the metabolomics processing software metaX, followed by PCA and Partial Least Squares-Discriminant Analysis (PLS-DA) to calculate the Variable Importance in Projection (VIP) value for each metabolite. Univariate analysis was performed using Student’s *t*-test to determine statistical significance (*P* value) between groups, and the fold change (FC) of metabolites was calculated. Differential metabolites were screened based on the default criteria: VIP>1, *P* < 0.05, and FC ≥ 2 or FC ≤ 0.5. Volcano plots were generated using the R package ggplot2, integrating the VIP value, log2(FC), and −log10(*P* value) to facilitate the identification of metabolites of interest. Clustered heatmaps were plotted using the R package pheatmap, with metabolite abundance normalized using the *z*-score. Correlation analysis between differential metabolites (using the Pearson correlation coefficient) was performed using the cor() function in R. Statistical significance was assessed using the cor.mtest() function, with *P* < 0.05 considered statistically significant. Correlation plots were visualized using the corrplot package, and bubble plots were generated using ggplot2. Finally, the KEGG database was utilized to investigate metabolite functions and metabolic pathways. A pathway was considered enriched when *x*/*n* > *y*/*N* and was deemed significantly enriched when the *P* value was <0.05.

### Statistical analysis

Unless otherwise indicated, data are presented as mean ± SEM from at least three independent biological replicates. Statistical significance was evaluated using unpaired Student’s *t*-tests using GraphPad Prism 10.1.2. *P* value <0.05 was considered statistically significant. Significance levels are denoted as *P* < 0.05, *P* < 0.01, *P* < 0.001, and *P* < 0.0001.

## Data Availability

The untargeted metabolomics and metagenomic sequencing data have been deposited in the National Microbiology Data Center under accession numbers NMDC10019825 and NMDC10019824. Any additional information required to reanalyze the data reported in this paper is available from the lead contact upon request. This paper does not report original code.

## SUPPLEMENTAL MATERIAL

Fig. S1 spectrum.03637-25-s0001.tiff Morphological characteristics of lyophilized powder CG-pp1 in the gastrointestinal tract at different time points post-ingestion, related to Fig. 3.

Fig. S2 spectrum.03637-25-s0002.tiff Data quality control for untargeted metabolomics of the Z8h and the negative group.

Fig. S3 spectrum.03637-25-s0003.tiff Cluster analysis of differential metabolites between the Z8h and the negative group.

Fig. S4 spectrum.03637-25-s0004.tiff KEGG purine metabolism pathway map of enriched differential metabolites in the CEF and LIF groups, related to Fig. 6B.

Supplemental material spectrum.03637-25-s0005.docx Supplemental figure legends.

ASM does not own the copyrights to Supplemental Material that may be linked to, or accessed through, an article. The authors have granted ASM a non-exclusive, world-wide license to publish the Supplemental Material files. Please contact the corresponding author directly for reuse.
