# Supplementary figures and images for: Formulation-dependent kinetics of Lacticaseibacillus paracasei Zhang in mice
Source: Microbiol Spectr. 2026 Apr 21;14(6):e03637-25. doi: 10.1128/spectrum.03637-25 (PMC13228022; doi:10.1128/spectrum.03637-25)

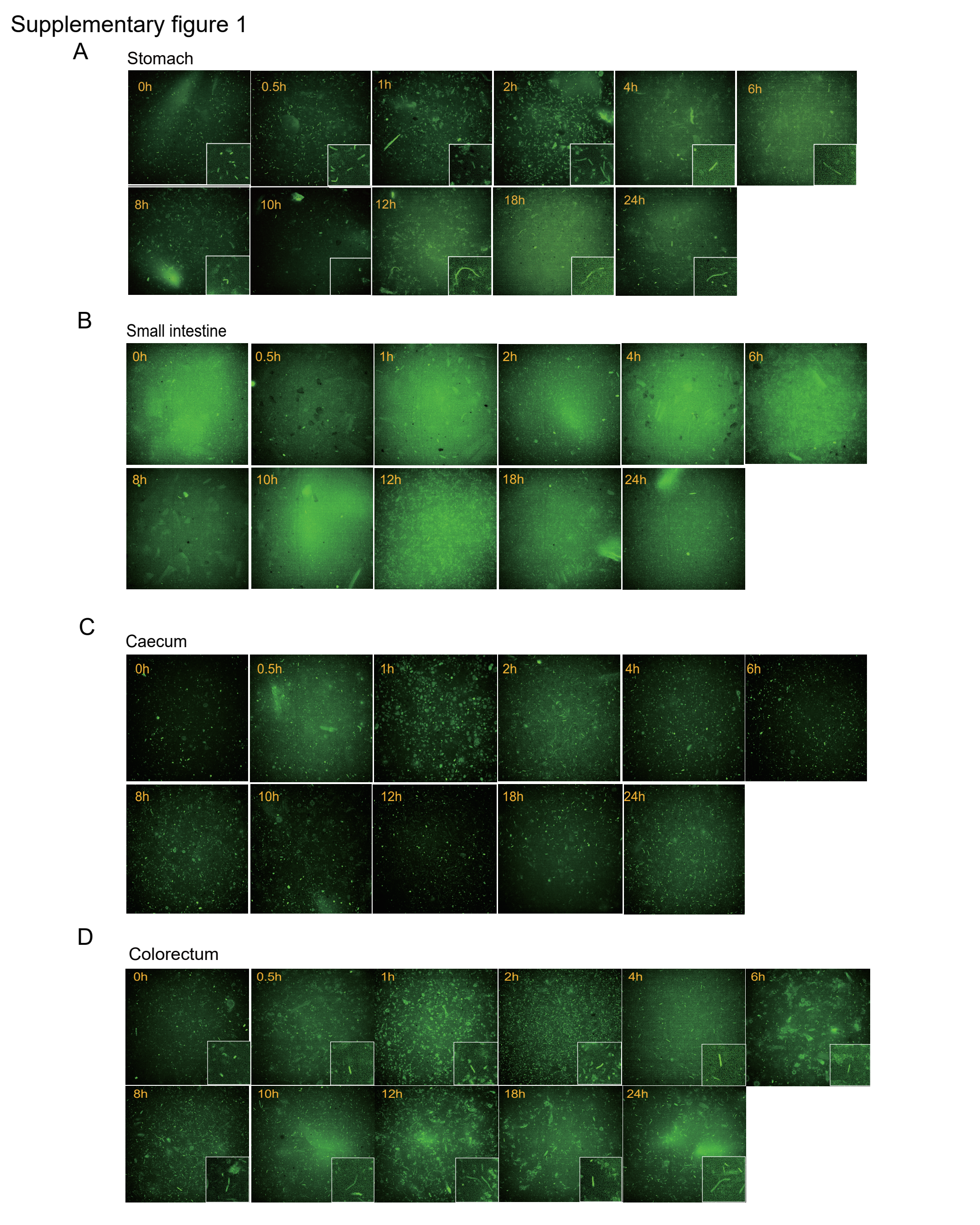

Supplement: Fig. S1 — Morphological characteristics of lyophilized powder CG-pp1 in the gastrointestinal tract at different time points post-ingestion, related to Fig. 3. [file spectrum.03637-25-s0001.tiff]

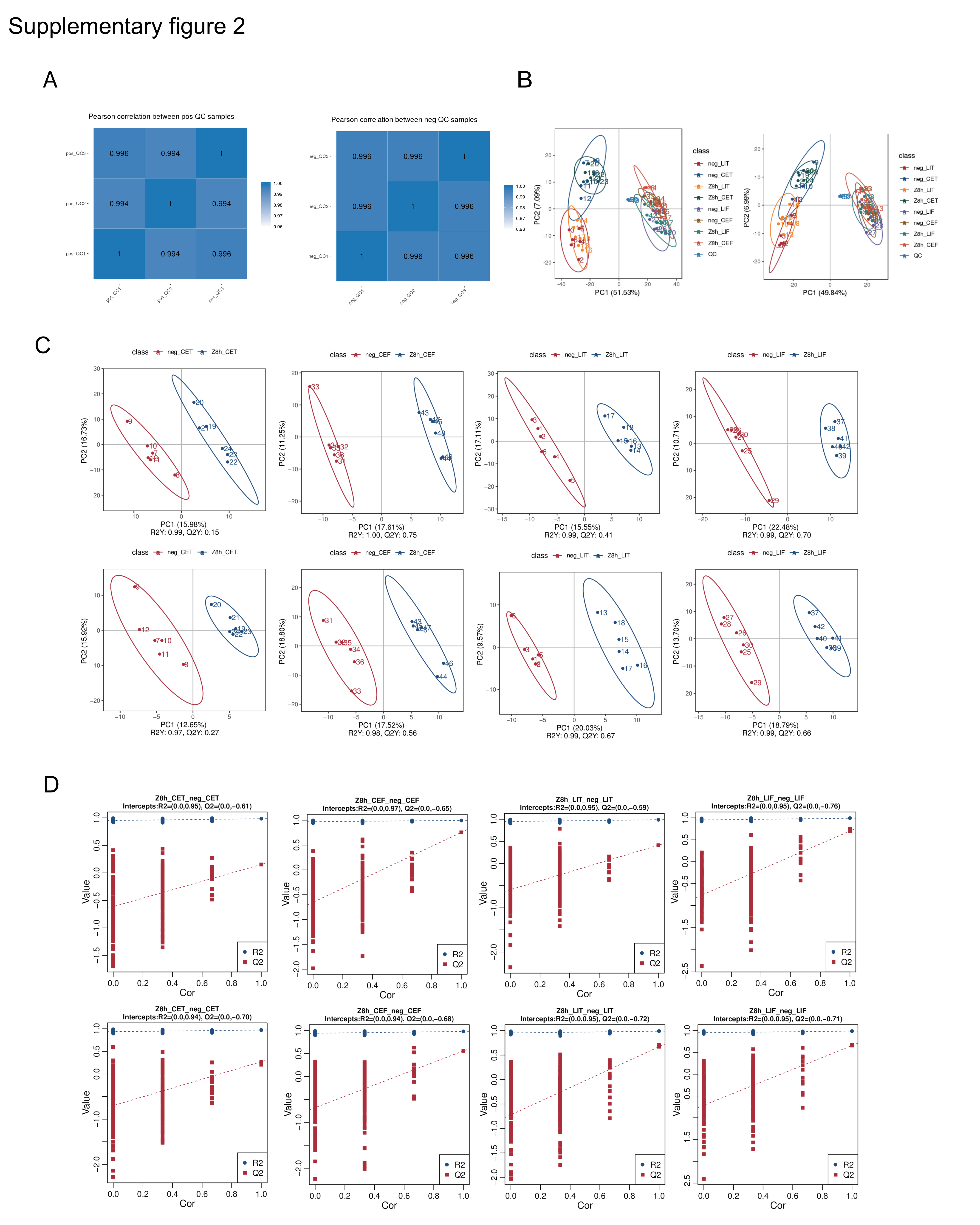

Supplement: Fig. S2 — Data quality control for untargeted metabolomics of the Z8h and the negative group. [file spectrum.03637-25-s0002.tiff]

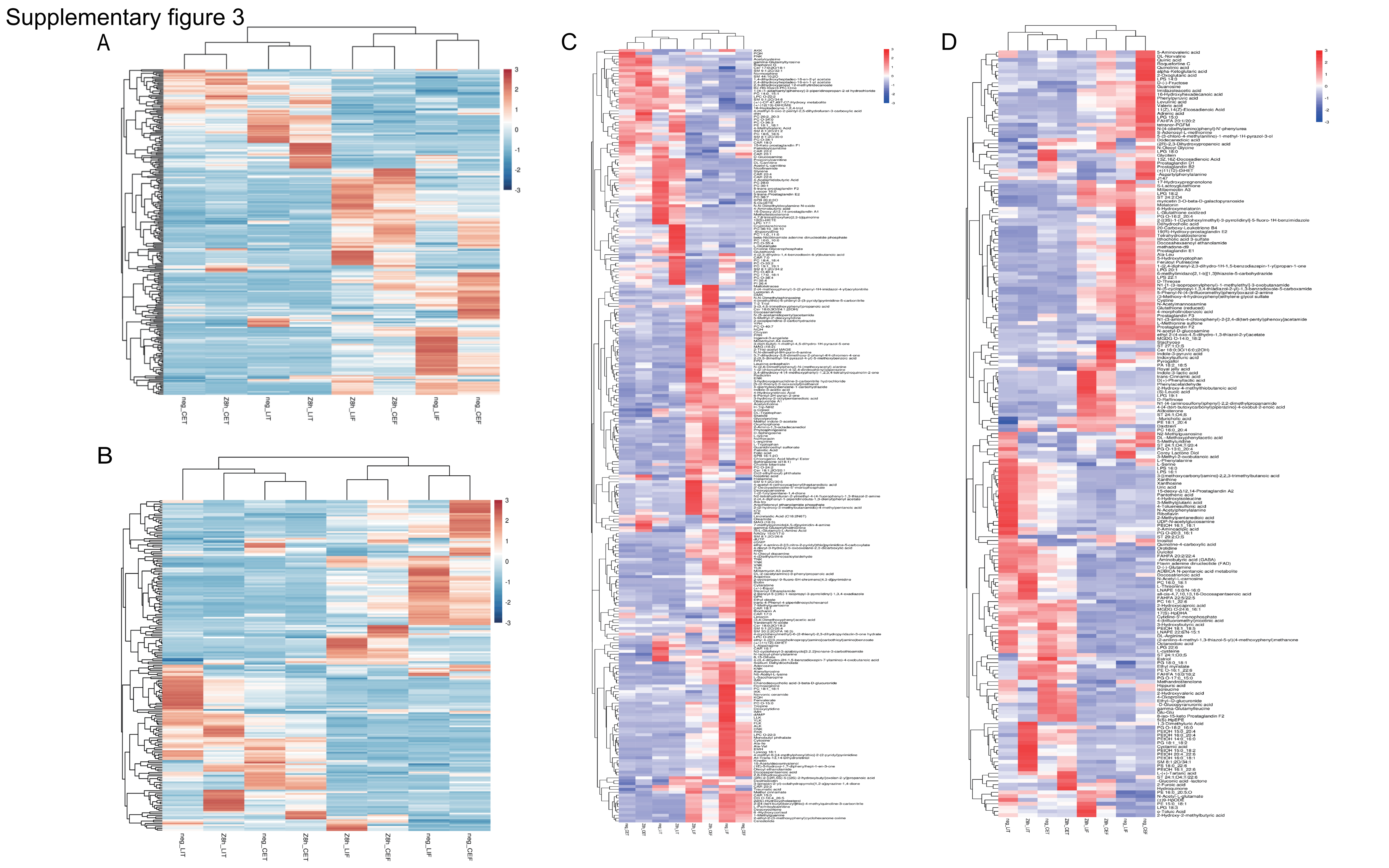

Supplement: Fig. S3 — Cluster analysis of differential metabolites between the Z8h and the negative group. [file spectrum.03637-25-s0003.tiff]

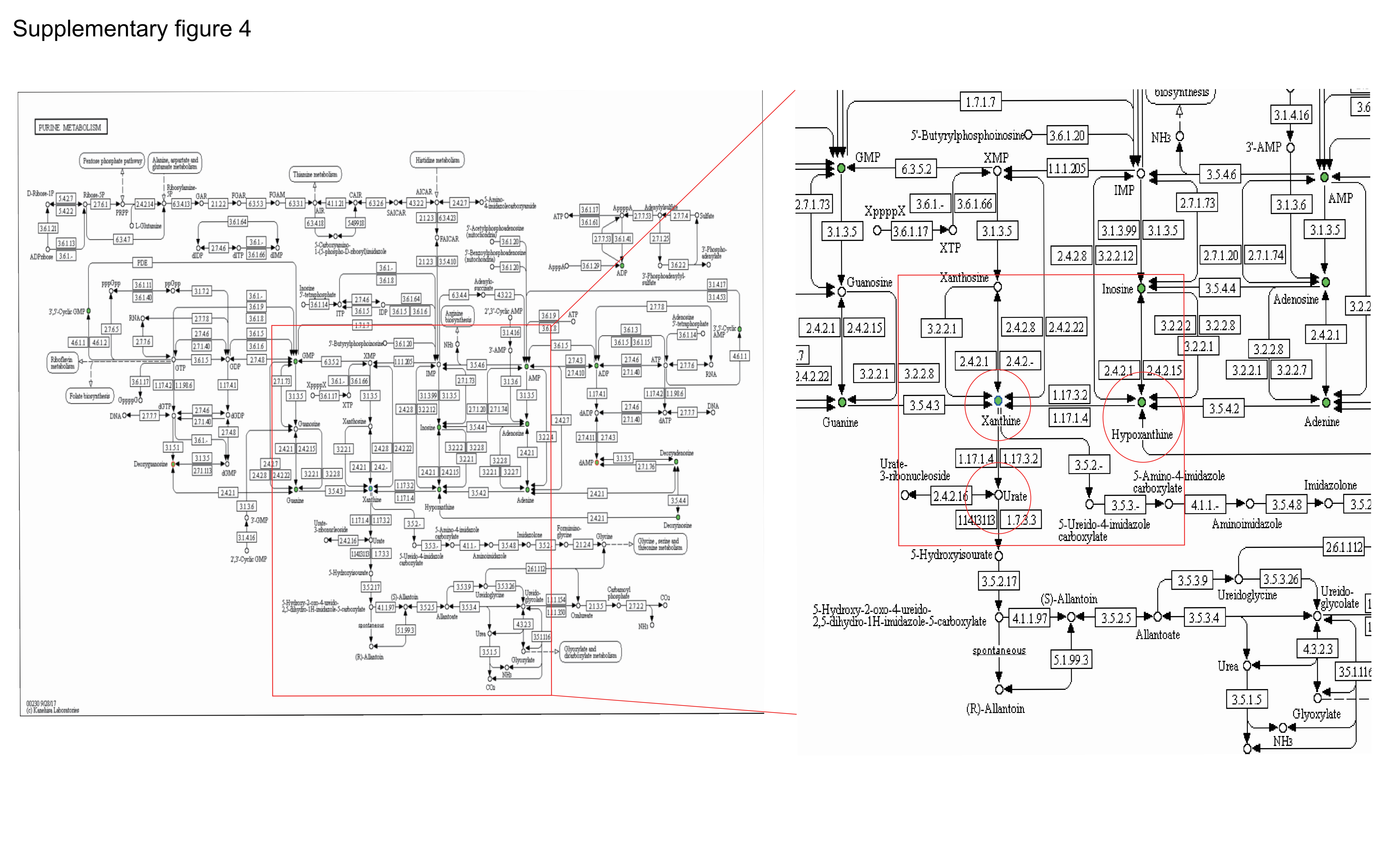

Supplement: Fig. S4 — KEGG purine metabolism pathway map of enriched differential metabolites in the CEF and LIF groups, related to Fig. 6B. [file spectrum.03637-25-s0004.tiff]
