# Supplementary material for: Formulation-dependent kinetics of Lacticaseibacillus paracasei Zhang in mice
Source: Microbiol Spectr. 2026 Apr 21;14(6):e03637-25. doi: 10.1128/spectrum.03637-25 (PMC13228022; doi:10.1128/spectrum.03637-25)
Supplement: Supplemental material — Supplemental figure legends. [file spectrum.03637-25-s0005.docx]

**Supplementary Figure Legends**

**Figure S1. Morphological characteristics of lyophilized powder CG-pp1 in the gastrointestinal tract at different time points post-ingestion., related to Figure 3**

(A-D) High Content Analysis System of lyophilized powder CG-pp1 morphology in the stomach (A), small intestine (B), cecum (C), and colorectum (D) at various time points.

**Figure S2. Data quality control for untargeted metabolomics of the Z8h and the negative group**

(A) Higher correlation coefficients among QC samples (with |r| closer to 1) indicate greater stability of the detection process and higher data quality.

(B) Principal component analysis (PCA) of all samples.

(C) PLS-DA score plot: the horizontal and vertical axes represent the scores of the samples on the first and second principal components, respectively. *R^2^* Y indicates the explanatory rate of the model, while *Q^2^* Y is used to evaluate the predictive ability of the PLS-DA model; a model is considered well-established when *R^2^* Y is greater than *Q^2^* Y. Permutation test: the horizontal axis represents the correlation between the permuted Y and the original Y, and the vertical axis represents the scores for *R^2^* and *Q^2^*. The top panel represents the positive ion mode, and the bottom panel represents the negative ion mode.

(D) PLS-DA valid plot: Permutation test of the PLS-DA model. The permutation test was used to validate the model’s reliability. The x-axis represents the correlation coefficient between the original and permuted Y labels, while the y-axis represents the *R^2^* (goodness of fit) and *Q^2^* (predictive ability) values. The intercept of *R^2^* and *Q^2^* with the y-axis indicates the extent of overfitting; a lower *Q^2^* intercept (< 0.05) suggests that the model is robust and not overfitted. The top panel represents the positive ion mode, and the bottom panel represents the negative ion mode.

**Figure S3. Cluster analysis of differential metabolites between the Z8h and the negative group**

(A, B) Cluster analysis of all differential metabolites. (A) Positive ion mode. (B) Negative ion mode. (C, D) Cluster analysis of detailed differential metabolites. (C) Positive ion mode. (D) Negative ion mode. Hierarchical clustering analysis was performed on all differential metabolites across comparison pairs. Relative quantification values were normalized and used for clustering. Samples are displayed vertically, while metabolites are displayed horizontally. Shorter dendrogram branches indicate higher similarity. Horizontal arrangement reflects the clustering patterns of metabolite abundance across groups.

**Figure S4. KEGG purine metabolism pathway map of enriched differential metabolites in the CEF and LIF groups, related to Figure 6B**

In the KEGG pathway map, circles represent metabolites. Green filled circles indicate annotated metabolites; red circles indicate upregulated differential metabolites; blue circles indicate downregulated differential metabolites; and yellow circles indicate metabolites with mixed regulation (both up and down).
